# Loop-mediated isothermal amplification coupled with a nanoparticle-based lateral biosensor for rapid, sensitive, and specific detection of *Talaromyces marneffei*

**DOI:** 10.3389/fmicb.2025.1661312

**Published:** 2025-11-04

**Authors:** Yuhong Zhou, Rui Ye, Yizhe Wang, Xue Zhao, Honglan Yu, Yu Wang

**Affiliations:** Department of Clinical Laboratory, The First People’s Hospital of Guiyang, Guiyang, China

**Keywords:** *Talaromyces marneffei*, loop-mediated isothermal amplification, lateral flow biosensor, diagnosis, limit of detection

## Abstract

**Introduction:**

Talaromyces marneffei (*T.marneffei*) is a pathogenic dimorphic fungus endemic in Southeast Asia and southern China. It can be found in the environment (soil and decaying wood) and may infect human hosts following the inhalation of spores. The disseminated infection of *T.marneffei* can be life-threatening without timely diagnosis and effective antifungal therapy.

**Methods:**

Herein, we proposed a simple, rapid, sensitive and visual point-of-care detection method for detecting *T.marneffei* using loop-mediated isothermal amplification combined with a nanoparticle-based lateral flow biosensor (*T.marneffei-*LAMP-LFB). A set of six primers was designed based on the internal transcribed spacer (ITS) region of the rRNA gene of *T.marneffei*.

**Results:**

The results of the *T.marneffei*-LAMP were visually reported by the biosensor within 2 min. A range of pathogenic organisms, including various fungus species, as well as several Gram-negative and Gram-positive bacterial species and viruses, were used to determine the analytical sensitivity and specificity of the assay. The optimal reaction condition for the *T.marneffei*-LAMP-LFB assay was 67 °C within 40 min. The *T.marneffei*-LAMP-LFB was able to detect up to 400 ag per test. No cross-reactions to non-*T.marneffei* strains were obtained. The entire procedure, including sample processing (15 min), LAMP reaction (40 min), and result reporting (within 2 min), could be completed within 60 min. Among 120 whole blood samples tested, 25 (20.83%) were *T.marneffei*-positive by *T.marneffei*-LAMP-LFB, with a diagnostic accuracy of 100% when compared to fungal culture assay.

**Conclusion:**

The *T.marneffei*-LAMP-LFB assay targeting the ITS region of the rRNA gene is a rapid, highly sensitive, specific, and straightforward diagnostic tool that may be suitable for use in point-of-care settings and basic medical facilities, especially in rural areas.

## Introduction

*Talaromyces marneffei* (*T.marneffei*) is a dimorphic fungus commonly found in patients with HIV-infected and immunocompromised (Tsang et al., 2019; Sun et al., 2021). *T.marneffei* occurs primarily in Southeast Asia, including Thailand, Vietnam, India, Laos, Malaysia, and China’s Guangxi, Guangdong, and other regions (Morris et al., 2024). In recent years, cases of *T.marneffei* infection have also been reported in non-endemic areas (Patassi et al., 2013; Narayanasamy et al., 2021). The exact route of transmission of *T.marneffei* is unknown, but inhalation of fungal spores through the respiratory tract is believed to be the most common mode of infection (Pruksaphon et al., 2022). Common manifestations of disseminated *T.marneffei* infection include recurrent fever, anemia, weight loss, skin lesions, hepatosplenomegaly, lymphadenopathy, and gastrointestinal and respiratory symptoms. Once *T.marneffei* infection becomes systematic, it can progress rapidly and may be life-threatening without timely and effective antifungal treatment (Liu et al., 2021). Consequently, rapid and accurate identification of *T.marneffei* at an early stage is crucial for effective disease prevention and control.

Traditional diagnostic methods for *T.marneffei* infection rely on culture or microscopy, which are time-consuming and have relatively low positive detection rates; however, these methods are still widely used in most laboratories (Tam et al., 2015). Consequently, additional time and effort are required for culture and identification in clinical settings. To enable early, rapid, and accurate identification of *T.marneffei*, rapid, more efficient, sensitive, and specific diagnostic methods need to be established.

Recently, developing loop-mediated isothermal amplification (LAMP) has significantly improved the detection of pathogenic microorganisms (Jiang et al., 2019; Li et al., 2023; Zhou et al., 2024). It has been successfully employed to detect various bacteria, fungi, viruses, parasites, and other pathogens due to its high specificity, rapidity, and simplicity (Pu et al., 2022; Phurijaruyangkun et al., 2024; Ahmadi et al., 2025). However, traditional methods for verifying LAMP amplification products, including hydroxy naphthol blue (HNB), SYBR Green, and agarose gel electrophoresis (Yang et al., 2024), struggle to distinguish specific amplification from nonspecific amplification, which can lead to misunderstanding of the results. To address these limitations, a visual, sensitive, and target-specific lateral flow biosensor (LFB) can be used to verify nucleic acid-labeled amplification products (Wang et al., 2022; Zhu et al., 2025). This method effectively overcomes the shortcomings of other LAMP amplification product verification methods, such as agarose gel electrophoresis, real-time turbidimetry, and visual indicators, and shortens the verification program time (1–2 min).

The study aimed to develop a rapid and visual detection assay for *T.marneffei* using LAMP coupled with LFB based on the target sequence and to validate its potential application in clinical samples.

## Materials and methods

### Reagents and apparatus

**Figure 1:**
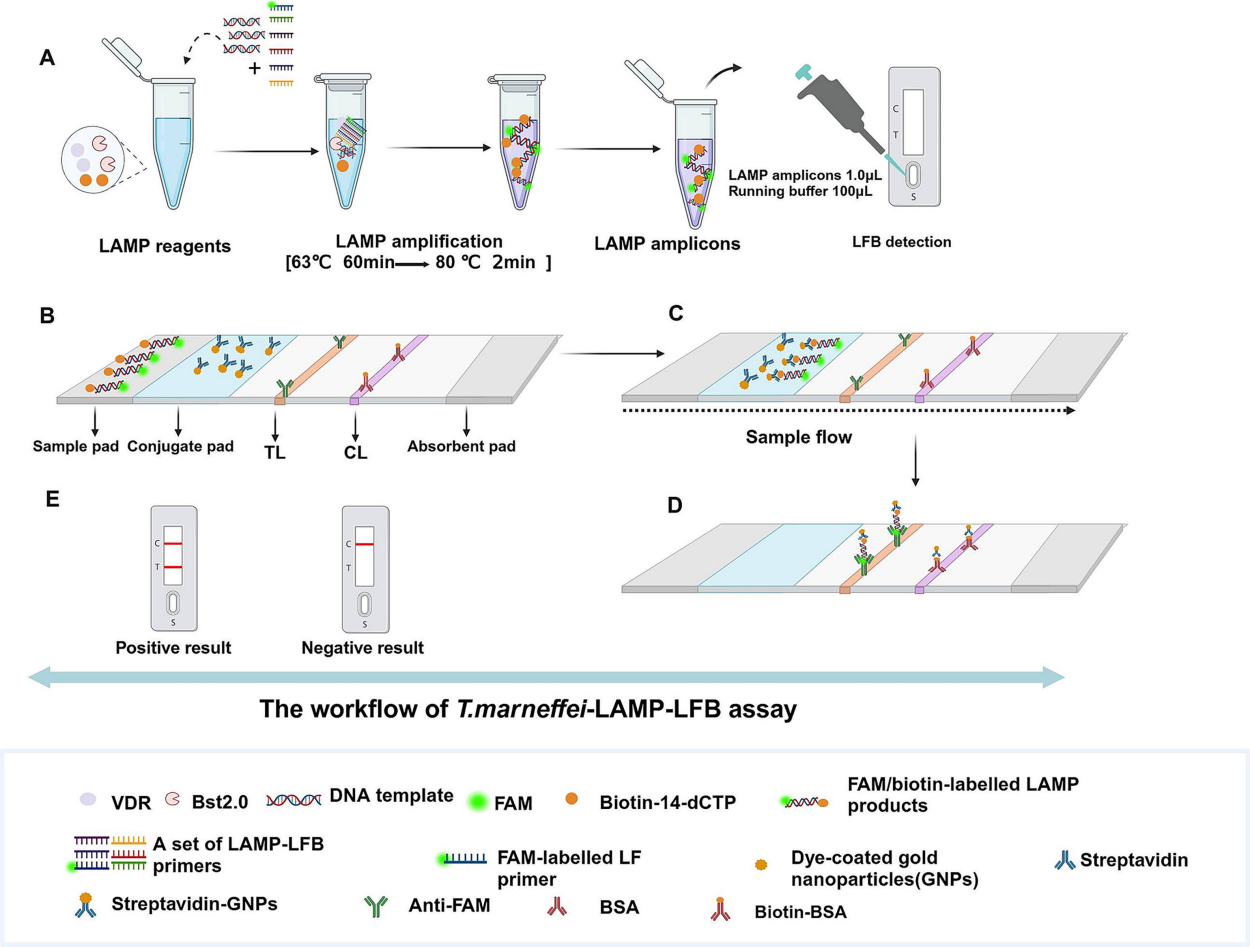
Schematic diagram of detection workflow of the *Talaromyces marneffei*-LAMP-LFB assay. **(A)** The *Talaromyces marneffei*-LAMP-LFB reaction system with premixed *Talaromyces marneffei* templates and isothermal reagents was incubated at 63 °C for 60 min and then terminated at 80 °C for 2 min and validation of *Talaromyces marneffei*-LAMP-LFB amplicons mediated by LFB biosensor. **(B)** *Talaromyces marneffei*-LAMP products (1.0 μL) and running buffer (100 μL) were simultaneously added to the sample pad. **(C)** The running buffer and *Talaromyces marneffei*-LAMP products move forward to conjugate pad and reaction region due to capillary action. **(D)** For positive results, the FAM/biotin-labeled LAMP products are arrested by anti-FAM at TL strip, and the streptavidin-GNPs are arrested through Biotin-BSA at CL strip. For negative results, only the streptavidin-GNPs flow to reaction region and arrested by Biotin-BSA at CL strip. **(E)** Interpretation of the *Talaromyces marneffei*-LAMP-LFB assay results: negative-only the CL appears on the LFB; positive-CL and TL appear on biosensor. LAMP, loop-mediated isothermal amplification; LFB, nanoparticle-based lateral biosensor; TL, test line; CL, control line.

Nucleic acid extraction kits for pathogens’ genomic DNA (Beijing Baitaike Biotech Co., Ltd., Beijing, China) and viral RNA (Tianlong Technology Co., Ltd., Xian, China) were commercially obtained. Isothermal amplification kits (RNA/DNA Universal) and visual detection reagents (VDR) were obtained from HUIDEXIN Biotech Co., Ltd. (Tianjin, China). The schematic design of gold nanoparticle-based LFB is shown in Figure 1. Briefly, the LFB consisted of four sections, namely, sample pad, conjugate pad, NC membrane with immobilized anti-FAM and biotin-BSA, and an absorbent pad, all of them were assembled on a plastic adhesive backing card (HuiDeXin Bio-technology, Tianjin, China). Dye coated gold nanoparticles (GNPs) were deposited on the conjugate pad, the size of GNPs was 35 ± 5 nm, and the concentration was 10 mg/mL. Rabbit anti-FAM antibody (0.2 mg/mL; Abcam) and biotin-BSA (4 mg/mL; Abcam) were fixed onto the NC membrane as test line (TL) and control line (CL), respectively. Each line was separated by 5 mm. The purity and concentration of the extracted nucleic acid were analyzed using a Nanodrop 2000 spectrophotometer (Thermo Fisher Scientific, Waltham, MA, USA) by measuring the A260/A280 ratio.

### Pathogenic bacteria and clinical samples

In this study, experimental bacteria, including 16 *T.marneffei* and 31 non-*T.marneffei-*isolated strains were used in Table 1. The genomic DNA templates were extracted using a DNA isolation kit (Bio TeKe, Beijing) according to the manufacturer’s instructions. Additionally, 120 clinical samples were collected from patients suspected of having *T.marneffei* infection who were admitted to *the First People’s Hospital of Guiyang*. To evaluate the clinical performance of *T.marneffei*-LAMP-LFB, all clinical samples were tested in parallel using both fungal culture and the *T.marneffei*-LAMP-LFB method. This study was approved by the ethics committee of *the First People’s Hospital of Guiyang* (Ethical approval No. G2024-S022). The extracted DNA was stored at −20 °C until use.

**Table 1:** Pathogens used in the current study.

Pathogen	Source of pathogens^1^	No. of strains	*Talaromyces marneffei*-LAMP-LFB result^2^
*Penicillium marnefei*	Isolated strains (GFPH)	16	P
*Staphylococcus aureus*	Isolated strains (GFPH)	1	N
*Klebsiella pneumoniae*	Isolated strains (GFPH)	1	N
*Haemophilus influenzae*	Isolated strains (GFPH)	4	N
*Enterococcus faecalis*	Isolated strains (GFPH)	4	N
*Escherichia coli*	Isolated strains (GFPH)	1	N
*Candida albicans*	Isolated strains (GFPH)	6	N
*Candida tropicalis*	Isolated strains (GFPH)	3	N
*Candida glabrata*	Isolated strains (GFPH)	3	N
*Candida parapsilosis*	Isolated strains (GFPH)	1	N
*Meyerozyma guilliermondii*	Isolated strains (GFPH)	1	N
*Pichia kudriavzevii*	Isolated strains (GFPH)	1	N
*Aspergillus flavus*	Isolated strains (GFPH)	1	N
*Listeria monocytogenes*	Isolated strains (GFPH)	1	N
*Norovirus*	Isolated strains (GFPH)	1	N
*Enterovirus A71* (EVA71)	Isolated strains (GZCDC)	1	N
*Coxsackievirus A16* (CVA16)	Isolated strains (GZCDC)	1	N

^1^GZCDC, Guizhou Provincial Center for Disease Control and Prevention; GFPH, *The First People’s Hospital of Guiyang*; ^2^P, positive; N, negative.

### Design of *Talaromyces marneffei*-LAMP-LFB primers

**Figure 2:**
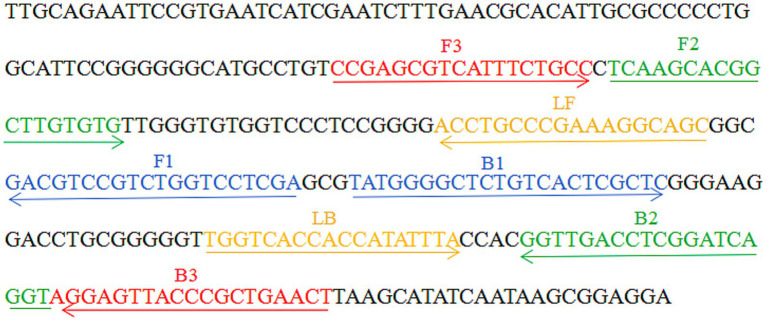
Appropriate primer design for *Talaromyces marneffei*-LAMP-LFB assay. The nucleotide sequences of the sense strand of the ITS region of the rRNA gene are listed. Right arrows and left arrows indicate sense and complementary sequences that are used.

A set of six primers, including two inner primers (FIP and BIP), two outer primers (F3 and B3), and two loop primers (LF and LB), were designed based on the reaction mechanism of LAMP to target the sequence of the internal transcribed spacer (ITS) region of the rRNA gene (Genbank accession No. AB353913.1). All primers were designed using Primer Explorer V5 (https://primer-explorer.jp/e/; Eiken Chemical Co., Ltd., Tokyo, Japan) and validated using the basic local alignment search tool (Wang et al., 2017). Figure 2 displays primer positions, while Table 2 depicts the primer sequences and modifications. All primers used in this study were synthesized by Beijing-Tsingke Biotechnology Co., Ltd. (Beijing, China) and were of HPLC purification grade.

**Table 2:** The primers used in the current study.

Primer/plasmid name^a^	Sequences and modifications	Length^b^	Gene
FIP	TCGAGGACCAGACGGACGTC-TCAAGCACGGCTTGTGTG	38 nt	ITS1
BIP	TATGGGGCTCTGTCACTCGCTC-ACCTGATCCGAGGTCAACC	41 nt	
LF	GCTGCCTTTCGGGCAGGT	18 nt	
LB	TGGTCACCACCATATTTAC	19 nt	
F3	CCGAGCGTCATTTCTGCC	18 nt	
B3	AGTTCAGCGGGTAACTCCT	19 nt	
LF*	5’-FAM-GCTGCCTTTCGGGCAGGT-3’	18 nt	

^a^LF*, 5’-labeled with FAM when used in the LAMP-LFB assay; ^b^nt, nucleotide. FAM, 6-carboxyfluorescein.

### The standard LAMP assay

The *T.marneffei*-LAMP-LFB amplification was conducted using a one-step, 25 μL reaction mixture containing the following components of 12.5 μL of 2 × reaction buffer, 1 μL of 2.0 Bst DNA polymerase, 1.6 μM each of FIP and BIP, 0.8 μM each of LF and LB, 0.4 μM each of F3 and B3, 1 μL of VDR indicator, 1 μL of biotin-14-dCTP, 1 μL of DNA template, and double distilled water (DW) was added to reach a final volume of 25 μL. The reaction tubes were incubated at 63 °C for 60 min, followed by enzyme inactivation at 80 °C for 2 min. The details of the standard LAMP reaction flow chart are depicted in Figure 1.

### *Talaromyces marneffei*-LAMP-LFB product detection

Three methods were employed to detect and verify the products of the *T.marneffei*-LAMP-LFB: real-time turbidity using the LA-500 (Eiken Chemical Co., Ltd., Japan), colorimetric indicators VDR and LFB. In the real-time turbidity method, a turbidity value greater than 0.1 was considered positive. The VDR method visually distinguished positive reactions by a color change from colorless to light green, while negative and blank control remained colorless. Finally, the LFB assay displayed positive results when both the control line (CL) and test line (TL) appeared simultaneously. Conversely, negative amplifications only indicated the CL.

### Optimization reaction temperature of the *Talaromyces marneffei*-LAMP-LFB

To optimize amplification efficiency, the reaction temperature of the *T.marneffei*-LAMP-LFB assay was evaluated across a temperature range of 61–68 °C in 1 °C increments. Amplification mixtures containing 1 μL DNA of *Candida albicans* served as negative control, while 1 μL of DW was a blank control. The assay was performed according to the standard LAMP assay, and amplification products from the *T.marneffei*-LAMP-LFB assay were monitored using the Real-time Turbidimeter LA-500.

### Sensitivity and optimal isothermal amplification time of the *Talaromyces marneffei*-LAMP-LFB assay

Genomic DNA templates from pure culture of *T.marneffei* were serially diluted 10-fold from 4 ng to 4 ag to confirm the limit of detection (LoD) and evaluate the analytical sensitivity of the LAMP-LFB assay. The LAMP-LFB reactions were conducted under the standard conditions described above, and the results were examined using a VDR and LFB. The LoD of the LAMP-LFB assay was verified as the last dilution of each positive test. Each dilution was tested at least three times to ensure reproducibility.

Additionally, the optimal reaction time for the *T.marneffei*-LAMP-LFB assay was determined by comparing four different incubation times (20, 30, 40, and 50 min). Amplification products generated at each time point were assessed using both LFB and VDR. Each reaction time was tested in triplicate to ensure consistency. In the experiments, 1 μL of DW was used as a blank control.

### Specificity of the *Talaromyces marneffei*-LAMP-LFB assay

To evaluate the specificity of the *T.marneffei*-LAMP-LFB assay, the genomic DNA including 16 *T.marneffei* and 31 non-*T.marneffei* isolated strains were tested under the optimal amplification temperature and time conditions (Table 1). Amplification products from all samples were monitored using LFB.

### Applicability of the *Talaromyces marneffei*-LAMP-LFB assay to clinical samples

To assess the feasibility of applying the *T.marneffei*-LAMP-LFB assay for clinical diagnostics, 120 blood samples were collected from patients suspected of *T.marneffei* infection at *the First People’s Hospital of Guiyang*. All blood samples were evaluated using both the *T.marneffei*-LAMP-LFB assay and the traditional clinical cultural-based. Routine laboratory diagnosis of *T.marneffei* included fungal culture and Gram staining. The *T.marneffei*-LAMP-LFB detection was performed as previously described. Online statistical software MedCalc (http://www.medcalc.org/calc/diagnostic_test.php) (Version 23.0.5) was used to analyze the traditional clinical cultural-based and *T.marneffei*-LAMP-LFB assay.

## Results

### Confirmation of *Talaromyces marneffei*-LAMP-LFB assay products

**Figure 3:**
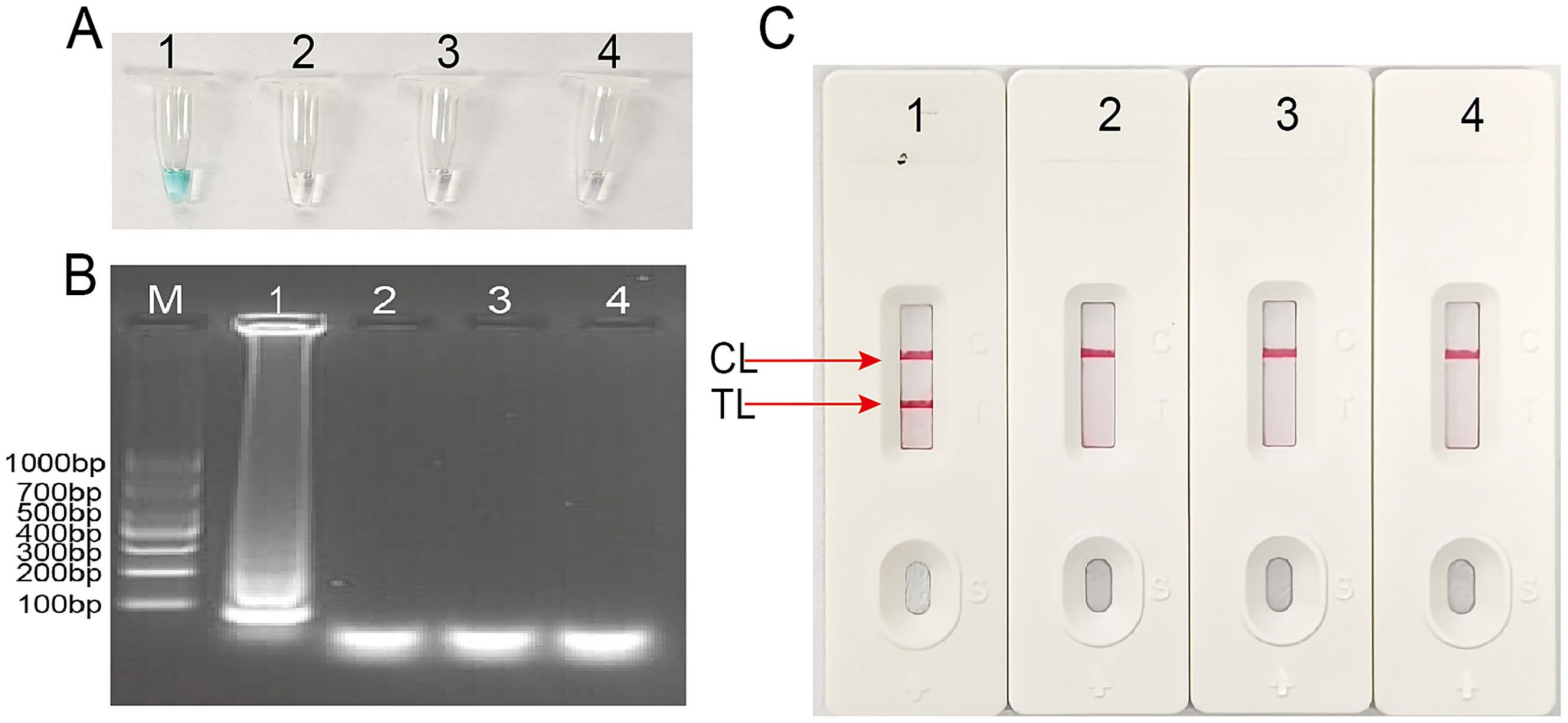
Identification and confirmation of *Talaromyces marneffei*-LAMP-LFB products. **(A)** The visible color changes of amplification products of *Talaromyces marneffei*-LAMP-LFB assay were analyzed by VDR Tube 1, positive amplification; tube 2, negative control DNA of *Candida albicans*; tube 3, negative control DNA of *Haemophilus influenzae*; tube 4, blank control (double-distilled water, DW). **(B)** Agarose gel electrophoresis applied to detecting *Talaromyces marneffei*-LAMP-LFB products. Lane BM, DL 1,000bp DNA markers, lane B1, positive amplification; lane B2, negative control DNA of *Candida albicans*; lane B3, negative control DNA of *Haemophilus influenzae*; lane B4, blank control (double-distilled water, DW). **(C)** The products of *Talaromyces marneffei*-LAMP-LFB were visually detected with lateral flow biosensor. Biosensor 1, positive amplification; Biosensor 2, negative control DNA of *Candida albicans*; Biosensor 3, negative control DNA of *Haemophilus influenzae*; Biosensor 4, blank control (double-distilled water, DW).

To assess the specificity and feasibility of the *T.marneffei* LAMP primers (Table 2), *T.marneffei* LAMP reactions were performed at a constant temperature at 63 °C for 60 min using *T.marneffei* DNA as the template. Amplification products were then visualized using three methods: VDR (Figure 3A), agarose gel electrophoresis (Figure 3B; Supplementary Figure S1) and LFB (Figure 3C). As illustrated in Figure 3, positive results were obtained only with *T.marneffei* nucleic acid, while no amplification was observed with *Candida albicans*, *Haemophilus influenzae*, or the blank control. These results demonstrate the validity of the *T.marneffei*-LAMP-LFB primers for the ITS region of the rRNA gene detection and their suitability for establishing the *T.marneffei*-LAMP-LFB assay.

### The optimal amplification temperature for the *Talaromyces marneffei*-LAMP-LFB assay

**Figure 4:**
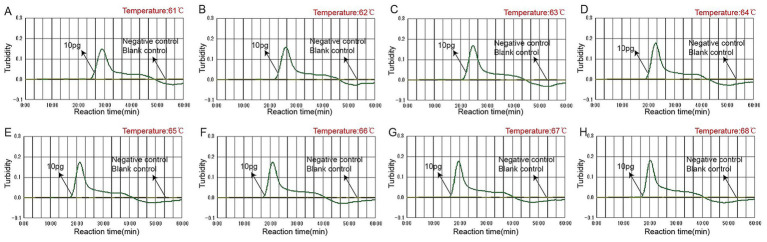
Optimal amplification temperature for *Talaromyces marneffei*-LAMP-LFB assay. By using a real-time measurement to monitor the turbidity of *Talaromyces marneffei*-LAMP-LFB reactions. The corresponding curves were displayed in the panels. Eight kinetic graphs **(A–H)** were generated at various temperatures (61–68 °C, 1 °C intervals) with target pathogens DNA at the level of 10 pg. per tube (The threshold value was 0.1 and the turbidity of >0.1 was considered to be positive). The graphs from **B** (62 °C) to **H** (68 °C) showed robust reaction. The optimal amplification temperature was 67 °C.

To achieve better amplification efficiency, the reaction temperature of the LAMP-LFB assays was optimized under standard conditions, and different incubation temperatures ranging from 61–68 °C with an interval of 1 °C were used for the LAMP assays. Real-time turbidity was monitored using an LA-500 instrument. Based on the results presented in Figure 4, 67 °C was identified as the optimal reaction temperature for *T.marneffei*-LAMP-LFB amplification. This conclusion is supported by the observation that the threshold value of 0.1 absorbance, indicative of positive amplification, was achieved most rapidly at 67 °C. Consequently, 67 °C was used for all subsequent *T.marneffei*-LAMP-LFB reactions in this study.

### Sensitivity and optimized time of the *Talaromyces marneffei*-LAMP-LFB assay

**Figure 5:**
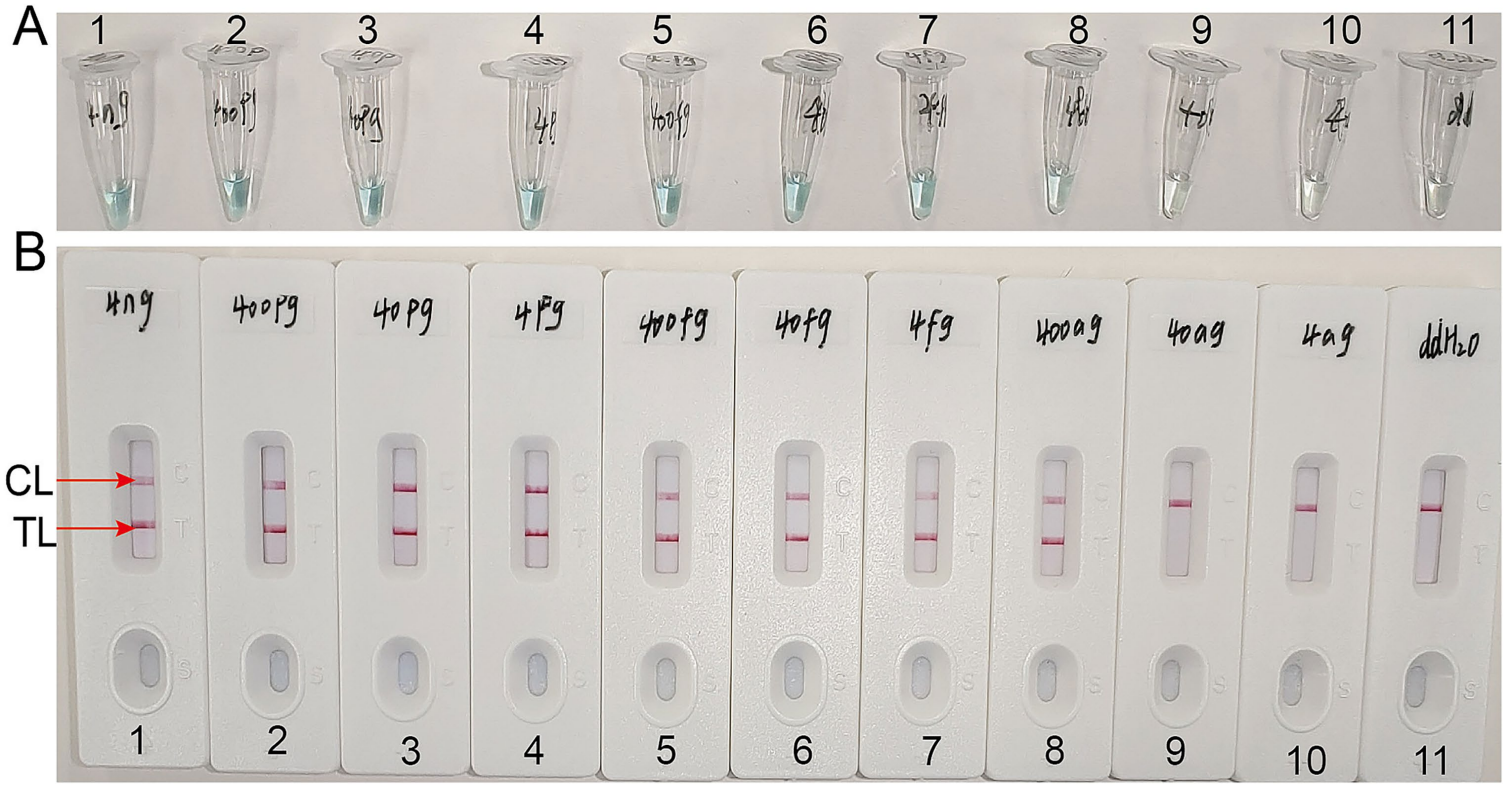
Sensitivity analysis of the *Talaromyces marneffei*-LAMP-LFB assays with serial dilutions of *Talaromyces marneffei* genomic DNA concentrations. Two measurement techniques, including **(A)** A colorimetric indicator (VDR) and **(B)** Lateral flow biosensor. A series of dilutions from 4 ng to 4 ag per microliter DNA and a blank control double-distilled water were operated according to standard LAMP-LFB reactions.

Using the LAMP-LFB method, the sensitivity of the *T.marneffei*-LAMP-LFB assay was tested through serial dilutions of extracted DNA. The final concentrations of the DNA templates were 4 ng, 400 pg., 40 pg., 4 pg., 400 fg, 40 fg, 4 fg, 400 ag, 40 ag, and 4 ag per reaction mixture. As presented in Figure 5, when the dilution exceeded 400 ag, the LAMP tubes were colorless and only displayed the control band on the LFB.

**Figure 6:**
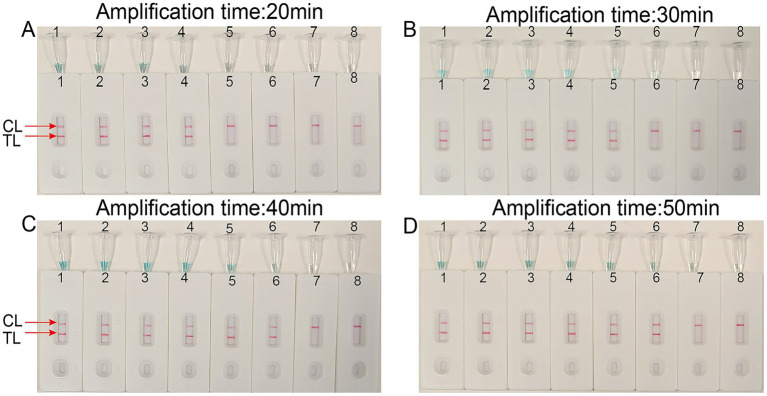
Optimal detection time required for *Talaromyces marneffei*-LAMP-LFB assay. Four different reaction times [**(A)** 20 min; **(B)** 30 min; **(C)** 40 min; **(D)** 50min] were evaluated at 67 °C. Biosensors 1–7 represent *Talaromyces marneffei* DNA levels of 40 pg. to 40 ag copies per reaction, respectively; 8 represents a blank control double-distilled water.

To determine the optimal amplification time for the *T.marneffei*-LAMP-LFB assay, four different incubation times (20, 30, 40, and 50 min) were evaluated at 67 °C. The amplified products were subsequently analyzed using LFB. The results confirmed that the LoD level of DNA (400 ag per reaction) was tested when the amplification lasted 40 min (Figure 6). Consequently, an amplification temperature of 67 °C and a duration of 40 min were selected as the optimal conditions for subsequent *T.marneffei*-LAMP-LFB assays in this study.

### Specificity of the *Talaromyces marneffei*-LAMP-LFB assay

**Figure 7:**
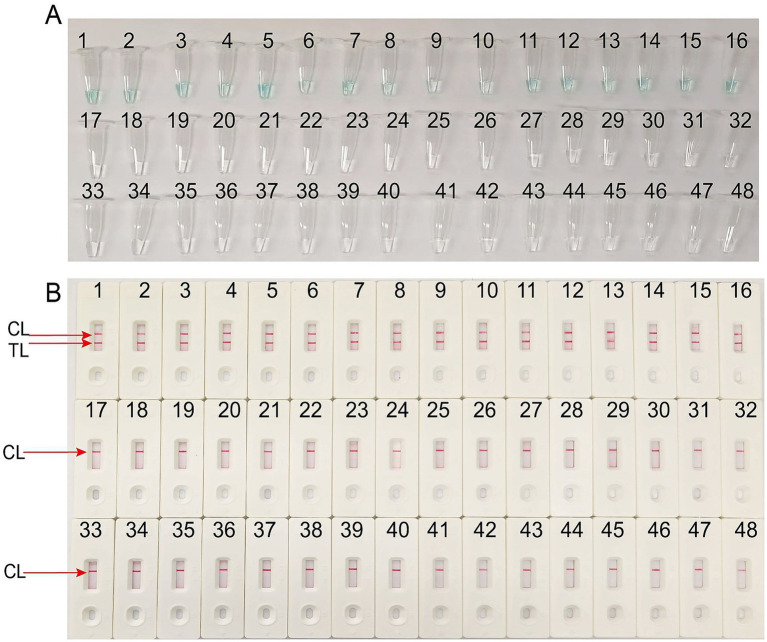
Specificity of LFB assays detecting *Talaromyces marneffei*-LAMP-LFB products. **(A)** The visible color changes of amplification products of *Talaromyces marneffei*-LAMP-LFB assay were analyzed by VDR. **(B)** The products of *Talaromyces marneffei*-LAMP-LFB were visually detected with LFB. The VDR visually distinguished positive reactions by a color change from colorless to light green, while negative and blank control remained colorless. Both the control line and the test line were visible in LFB for all *Talaromyces marneffei*, and only the control line was appeared in non-*Talaromyces marneffei*. 1–16, *Talaromyces marneffei* isolated strains; 17, *Staphylococcus aureus* isolated strains; 18, *Klebsiella pneumoniae* isolated strains; 19–22, *Haemophilus influenzae* isolated strains; 23–26, *Enterococcus faecalis* isolated strains; 27, *Escherichia coli* isolated strains; 28–33, *Candida albicans* isolated strains; 34–36, *Candida tropicalis* isolated strains; 37–39, *Candida glabrata* isolated strains; 40, *Candida parapsilosis* isolated strains; 41, *Meyerozyma guilliermondii* isolated strains; 42, *Pichia kudriavzevii* isolated strains; 43, *Aspergillus flavus Aspergillus flavus*; 44, *Listeria monocytogenes* isolated strains; 45, *Norovirus* clinical samples; 46, *EVA71* isolated strains; 47, *CVA16* isolated strains; 48, a blank control double distilled water (DW).

The specificity of the *T.marneffei*-LAMP-LFB assay was evaluated using a panel of 16 isolated *T.marneffei* strains and 31 non-*T.marneffei* pathogens, as detailed in Table 1. The *T.marneffei*-LAMP-LFB detection assays were performed under the previously established optimal conditions (67 °C and 40 min incubation). As illustrated in Figure 7, samples containing *T.marneffei* exhibited a color change from colorless to light green and produced two distinct red lines at both the TL and CL positions on the LFB strip, while all non-*T.marneffei* pathogens and the blank control remained colorless and indicated only a single red line at the CL position. These results confirm that the *T.marneffei*-LAMP-LFB assay can accurately differentiate *T.marneffei* from other microbes.

### Application of LAMP-LFB to *Talaromyces marneffei* clinical samples

To assess the clinical applicability of the *T.marneffei*-LAMP-LFB assay, 120 clinical blood samples from patients suspected of *T.marneffei* were simultaneously tested using both the *T.marneffei*-LAMP-LFB and a traditional fungal culture method. Compared with traditional fungal culture assay, our assay’s sensitivity and specificity were 100% [95% confidence interval (CI): 86.28–100.00%] and 100% (95% CI: 96.19–100.00%), respectively (Table 3).

**Table 3:** Comparison of culture and *Talaromyces marneffei*-LAMP-LFB methods to identify *Talaromyces marneffei* in clinical samples.

*Talaromyces marneffei*-LAMP-LFB	Fungal culture method			Sensitivity		Specificity	
	Positive	Negative	Total	Value	95%CI	Value	95%CI
Positive	25	0	25	100.00%	86.28% to 100.00%	100.00%	96.19% to 100.00%
Negative	0	95	95				
Total	25	95	120				

## Discussion

*T.marneffei* is the only temperature-bidirectional fungus in the Penicillium genus and is the causative agent of disseminated and progressive infection in humans, primarily in Southeast Asian countries and southern China (Wang et al., 2023). Approximately 17,300 cases of *T.marneffei* infection are diagnosed each year, with a reported mortality rate of extremely high (~1/3) (Wang et al., 2023). In recent years, with the increasing incidence of HIV, the widespread use of organ transplantation and various catheter interventions, and the extensive use of broad-spectrum antibiotics, adrenocortical hormones, and immunosuppressants, the immunity of many patients has been deficient or declining. The incidence of *T.marneffei* infection has increased significantly, and the epidemic area has been continuously expanding (Casalini et al., 2024). Patients infected with *T.marneffei* are often misdiagnosed with other infections, particularly tuberculosis or bacterial pneumonia, due to overlapping clinical features and similar pulmonary imaging findings (Harika et al., 2024).

Currently, delayed diagnosis is the primary cause of a high mortality rate in patients co-infected with HIV or infected with *T.marneffei* (Ye et al., 2024). *T.marneffei* is typically diagnosed through microscopic identification and culture techniques of the fungal culture in clinical samples. Due to its unique morphological and dimorphic characteristics, *T.marneffei* grows as a mycelium at 25 °C and as yeast at 37 °C (Cao et al., 2007). However, traditional detection methods (microbial culture, histopathological examination, and serological determination) usually cannot meet the requirements for rapid detection in terms of time and sensitivity.

Consequently, there is a critical need for rapid, accurate, and sensitive diagnostic tools or kits to facilitate prompt clinical intervention and effective disease control. Molecular biology methods and techniques have significantly improved the early and rapid diagnosis of infectious diseases. Over the years, several nucleic acid-based assays have been developed for *T.marneffei* detection (nested or semi-nested PCR, PCR enzyme immunoassays, and PCR hybridization); however, these methods are often limited by their high cost, routine operation, and applicability in laboratories lacking specialized equipment (Liu et al., 2016; Lu et al., 2016). Among the various rapid testing methods, LAMP is an innovative gene amplification method that provides a rapid and straightforward tool for the early detection and identification of microbial pathogens. It has been widely used for the detection of various pathogens, including viruses, bacteria, and fungi (Notomi et al., 2015). However, the validation and analysis of LAMP products, including VDR, agarose gel electrophoresis, and real-time turbidimetry, remains a key challenge.

The LFB was designed and developed to validate LAMP amplification products by labeling special primers, and it is widely used in the analysis of LAMP products (Chen et al., 2021; Jiang et al., 2021). During operation, only (1.0 μL) of the LAMP amplicon and the same (100 μL) of the running buffer were added to the sample pad, and the results were verified by observing the color changes in TL and/or CL with the naked eye. Using LFB (1–2 min) greatly reduces the analysis time of LAMP products. Compared with other methods (colorimetric index, agarose gel electrophoresis, real-time turbidity), the LFB method is not only fast and sensitive but also a user-friendly, and error-tolerant approach, especially in terms of reducing the need for special instruments, reagents, and other verification steps.

In the present study, a set of primers was designed based on the ITS region of the rRNA gene of *T.marneffei*, and the gene was amplified via an isothermal amplification device. The clinical effectiveness of ITS targets has been confirmed in multiple diagnostic studies, especially suitable for the early detection of yeast-like infections, providing a basis for the recognition of *T. marneffei* (Liu et al., 2016; Lu et al., 2016; Wang et al., 2025). To determine the best reaction conditions, we used different reaction temperatures and reaction times in the LAMP-LFB assay. The amplification efficiency of LAMP-LFB at 67 °C was greater than that at other temperatures (Figure 4). Additionally, *T.marneffei*-LAMP-LFB has high sensitivity, and the results revealed that the LoD level of the genomic DNA 400 ag per microliters could be detected after 40 min of amplification (Figures 5, 6). It has a higher sensitivity than currently applied molecular methods, which are 10–100 times greater than those of nested PCR and real-time PCR (Liu et al., 2016; Lu et al., 2016). In addition to the high sensitivity and high specificity of the *T.marneffei*-LAMP-LFB assay, only *T.marneffei* samples were detected, while other species were undetected (Figure 7). To verify its practicality, a comparative method was used to detect *T.marneffei*-LAMP-LFB in 120 clinical blood samples, and the results also revealed that *T.marneffei*-LAMP-LFB was less time-consuming, consistent with the results of traditional fungal culture methods (Table 3).

A limitation of our study is that we only tested the assay on patient blood samples. The clinical diversity of *T.marneffei* infection can include damage to the skin, mouth, lungs, digestive tract, bone, and other systems, and our method of examination also requires different types of clinical samples. Although there are limitations, further research is needed to validate its clinical application, and the apparent advantage of this method lies in its speed and high specificity. In this experiment, LFB is more convenient, accurate and operable regarding instrument requirements. As *T.marneffei*-LAMP-LFB only requires incubation at an isothermal temperature of 67 °C for 40 min and does not require sophisticated instrumentation, many thermostatically controlled instruments can be used for amplification procedures (constant-temperature water baths), making it particularly suitable for care sites and field laboratories.

## Conclusion

In conclusion, we developed a novel method (*T.marneffei*-LAMP-LFB) for timely, portably and accurately diagnosis of *T.marneffei* infection. The *T.marneffei*-LAMP-LFB method could intuitively reflect the detection result and eliminate the need for complex procedures and costly instrumentation. In the process of application and evaluation, the *T.marneffei*-LAMP-LFB method indicated good specificity and sensitivity for detecting *T.marneffei* samples within 1h with simple thermostatic instruments. It can be considered a potential screening tool for *T.marneffei* infection in clinical, basic, field, and resource-poor areas.

## Data Availability

The datasets presented in this article are not readily available because the original contributions presented in the study are included in the article/Supplementary material. Further inquiries can be directed to the corresponding author. Requests to access the datasets should be directed to wangzhongyuwy@163.com.
