# Supplementary material for: Loop-mediated isothermal amplification coupled with a nanoparticle-based lateral biosensor for rapid, sensitive, and specific detection of Talaromyces marneffei
Source: Front Microbiol. 2025 Nov 4;16:1661312. doi: 10.3389/fmicb.2025.1661312 (PMC12623345; doi:10.3389/fmicb.2025.1661312)
Supplement: Supplementary file 1 [file Data_Sheet_1.docx]

**Supplementary material**

**
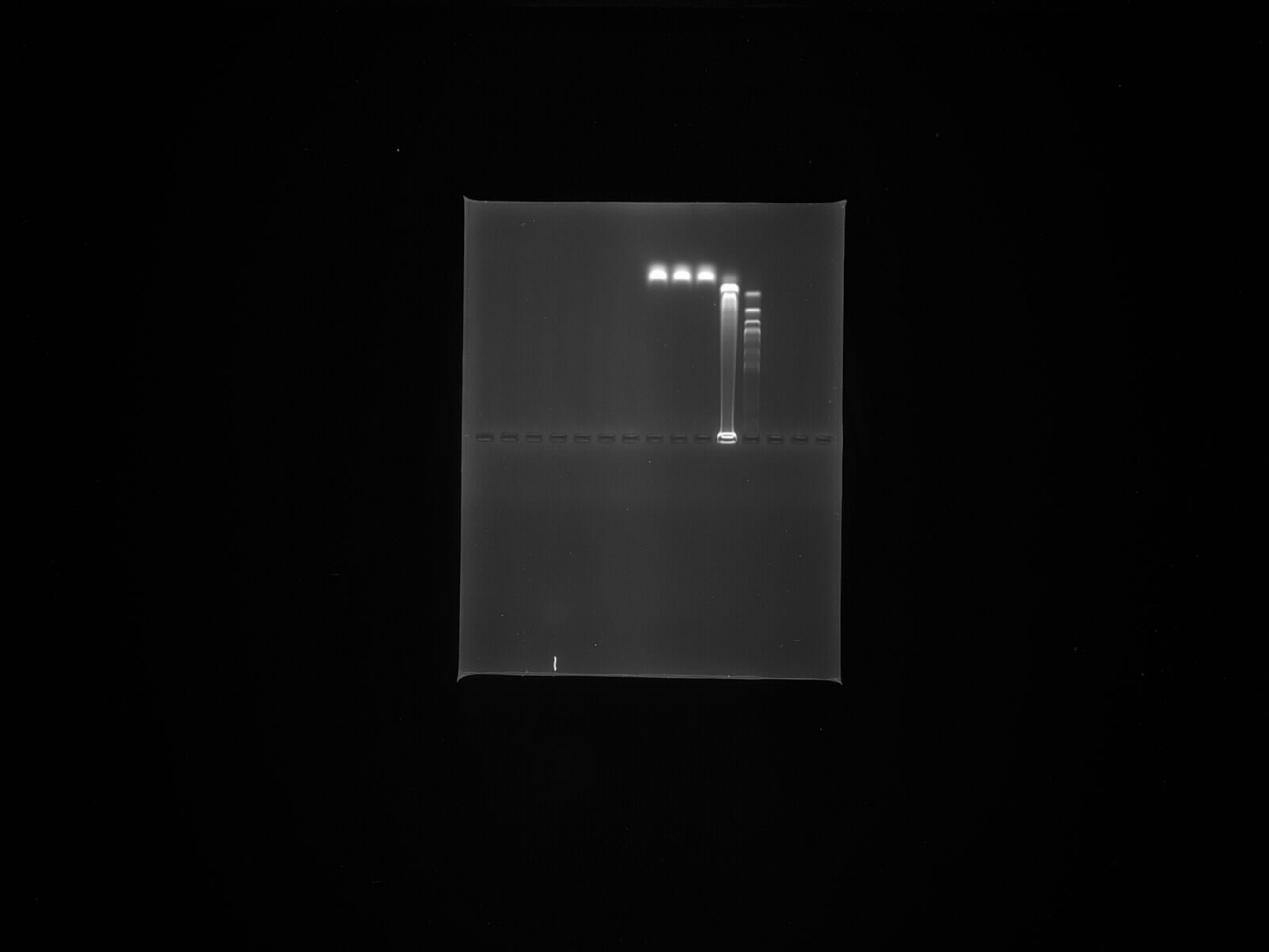
**

**Figure S1.** Agarose gel electrophoresis applied to detecting *T.marneffei*-LAMP-LFB products.
